# Supplementary material for: Distinguishing individual photobodies using Oligopaints reveals thermo-sensitive and -insensitive phytochrome B condensation at distinct subnuclear locations
Source: Nat Commun. 2024 Apr 29;15:3620. doi: 10.1038/s41467-024-47789-1 (PMC11058242; doi:10.1038/s41467-024-47789-1)
Supplement: Supplementary file 1 — Supplementary Information [file 41467_2024_47789_MOESM1_ESM.pdf]

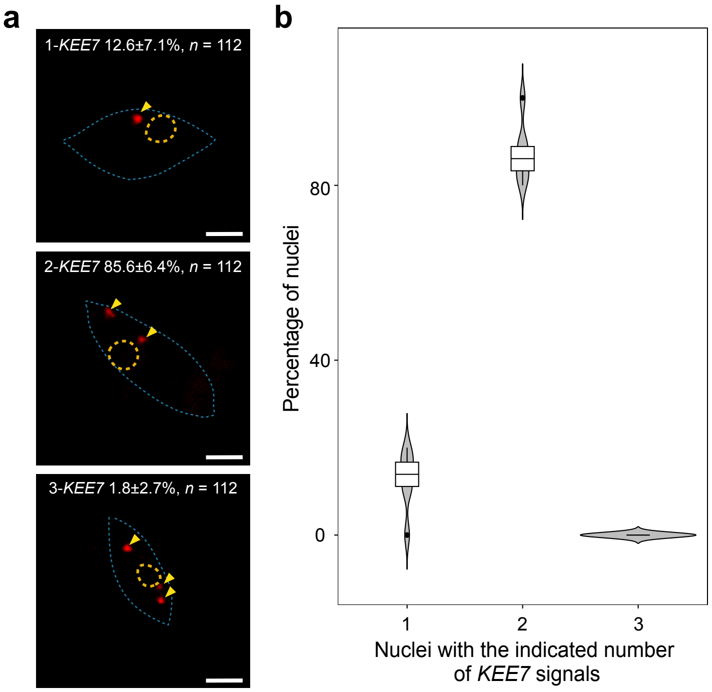

Supplementary Fig. 1. Sister chromatids are mostly aligned at the *KEE7* locus in pavement epidermal cells. a Confocal images showing *KEE7* FISH signals in pavement-cell nuclei from the cotyledons of 4-d-old *PBC* seedlings grown under 10 μmol m^{-2} s^{-1} R light at 16°C. *KEE7* was labeled via Oligopaint FISH (red) and indicated by orange arrowheads. Representative nuclei containing one to three *KEE7* signals are shown. The numbers indicate the percentage of each type of nuclei and the s.e. among the total number of observed nuclei (*n*). The boundaries of the nucleus and nucleolus are traced by dashed blue and orange lines, respectively. Scale bars are equal to 2 μm. **b** Violin plots showing the percentage of nuclei containing one, two, or three *KEE7* FISH signals. In the box and whisker plots, the boxes represent the 25% to 75% percentiles, the bars are equal to the mean.The source data underlying the PB quantifications in **b** are provided in the Source Data file.

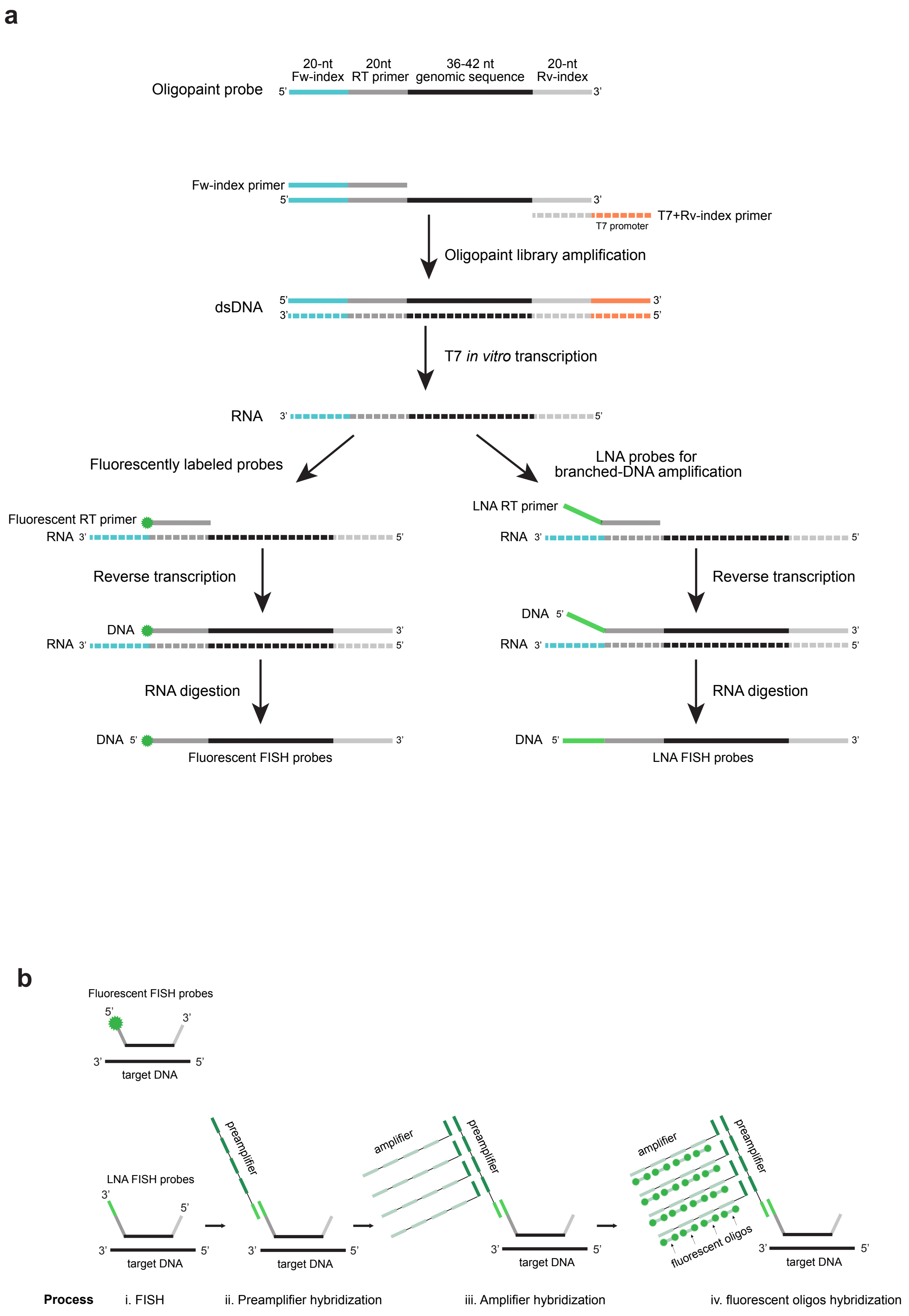

Supplementary Fig. 2. Oligopaint FISH probe synthesis.

Supplementary Table 1. List of fluorescently labeled reverse transcription primers for labeling *CEN178* probes.

Name	Sequence 5' - 3'
Fluorescent RT primer_Alex488	/5Alex488N/ACGTTCTGCGATCGGTCTGGA
Fluorescent RT primer_ATTO565	/5ATTO565N/ACGTTCTGCGATCGGTCTGGA
Fluorescent RT primer_ATTO633	/5ATTO633N/ACGTTCTGCGATCGGTCTGGA

Supplementary Table 2. List of oligo sequences used in the branched-DNA amplification of Oligopaint FISH. For LNA oligos, the LNA residues are labeled with a “+” sign preceding their positions.

Name	Sequence 5' - 3'
RT primer_488	TGCTGAGTGTCGCACGTTCTGCGATCGGTCTGGA
Preamplifier_488	TGCGA+C+ACTC+AGC+ATTTTTTTTTTGCTAGCCTATCAGATTTGCTAGCCTATCAGATTTGCTAGCCTATCAGATTTGCTAGCCTATCAGA
Amplifier_488	TC+TG+ATA+GGCTAGCTTTTTTTTTTCGCTATCCAGTGACTTTCGCTATCCAGTGACTTTCGCTATCCAGTGACTTTCGCTATCCAGTGAC
Fluorescent oligo_ATTO488	/5ATTO488N/AG+TCACTGG+A+TAG+CGA/3ATTO488N/
RT primer_565	CGCTGACGTACGGTCGTTCTGCGATCGGTCTGGA
Preamplifier_565	A+CCGTACGT+CAG+CGTTTTTTTTTTTAGCTAGCGCGTGTTTTTAGCTAGCGCGTGTTTTTAGCTAGCGCGTGTTTTTAGCTAGCGCGTGT
Amplifier_565	A+C+ACGCGCTAGCT+ATTTTTTTTTTGATCCGAGTATAGCTTTGATCCGAGTATAGCTTTGATCCGAGTATAGCTTTGATCCGAGTATAGC
Fluorescent oligo_ATTO565	/5ATTO565N/AGCT+ATAC+TC+GG+ATC/3ATTO565N/
RT primer_647	GCGCGTATATCAGCCGTTCTGCGATCGGTCTGGA
Preamplifier_647	GC+T+GATA+T+ACG+CGCTTTTTTTTTTCGTCGCCATAGTGTTTTCGTCGCCATAGTGTTTTCGTCGCCATAGTGTTTTCGTCGCCATAGTGT
Amplifier_647	AC+AC+TATG+GCGA+CGTTTTTTTTTTGTGCGCTCTCTCGATTTGTGCGCTCTCTCGATTTGTGCGCTCTCTCGATTTGTGCGCTCTCTCGA
Fluorescent oligo_ATTO647	/5ATTO647N/TCG+AG+AG+AGCGCAC/3ATTO647N/
